# Complementarity of Photo-Biomodulation, Surgical Treatment, and Antibiotherapy for Medication-Related Osteonecrosis of the Jaws (MRONJ)

**DOI:** 10.3390/medicina57020145

**Published:** 2021-02-05

**Authors:** Diana Florina Nica, Mircea Riviș, Ciprian Ioan Roi, Carmen Darinca Todea, Virgil-Florin Duma, Cosmin Sinescu

**Affiliations:** 1 Department of Anaesthesiology and Oral Surgery, School of Dental Medicine, “Victor Babes” University of Medicine and Pharmacy of Timisoara, 2A Eftimie Murgu Place, 300041 Timisoara, Romania; nica.diana@umft.ro (D.F.N.); ciprian.roi@umft.ro (C.I.R.); 2 Department of Oral Rehabilitation and Dental Emergencies, School of Dental Medicine, “Victor Babes” University of Medicine and Pharmacy of Timisoara, 2A Eftimie Murgu Place, 300041 Timisoara, Romania; todea.darinca@umft.ro; 3 3OM Optomechatronics Group, Faculty of Engineering, “Aurel Vlaicu” University of Arad, 2 Elena Dragoi Str., 310177 Arad, Romania; 4 Doctoral School, Polytechnic University of Timisoara, 1 Mihai Viteazu Ave., 300222 Timisoara, Romania; 5 Research Center in Dental Medicine Using Conventional and Alternative Technologies, School of Dental Medicine, “Victor Babes” University of Medicine and Pharmacy of Timisoara, 9 Revolutiei 1989 Ave., 300070 Timisoara, Romania; minosinescu@gmail.com

**Keywords:** medication-related osteonecrosis of the jaws (MRONJ), photo-biomodulation (PBM), surgical therapy, laser therapy, piezo-surgery, plasma rich fibrin (PRF)

## Abstract

*Background and Objectives*: Antiresorptive or anti-angiogenic agents may induce medication-related osteonecrosis of the jaws (MRONJ), which represents a challenge for clinicians. The aim of this study is to design and apply a composed and stage-approach therapy combining antibiotherapy, surgical treatment, and photo-biomodulation (PBM) for the prevention or treatment of MRONJ lesions. *Materials and Methods*: The proposed treatment protocol was carried out in the Department of Oral & Maxillofacial Surgery of the “Victor Babes” University of Medicine and Farmacy of Timisoara, in 2018–2020. A total of 241 patients who were previously exposed to antiresorptive or anti-angiogenic therapy, as well as patients already diagnosed with MRONJ at different stages of the disease were treated. A preventive protocol was applied for patients in an “at risk” stage. Patients in more advanced stages received a complex treatment. *Results:* The healing proved to be complete, with spontaneous bone coverage in all the *n* = 84 cases placed in an “at risk” stage. For the *n* = 49 patients belonging to stage 0, pain reductions and decreases of mucosal inflammations were also obtained in all cases. For the *n* = 108 patients proposed for surgery (i.e., in stages 1, 2, or 3 of MRONJ), a total healing rate of 91.66% was obtained after the first surgery, while considering the downscaling to stage 1 as a treatment “success”, only one “failure” was reported. This brings the overall “success” rate to 96.68% for a complete healing, and to 99.59% when downscaling to stage 1 is included in the healing rate. *Conclusions:* Therefore, the clinical outcome of the present study indicates that patients with MRONJ in almost all stages of the disease can benefit from such a proposed association of methods, with superior clinical results compared to classical therapies.

## 1. Introduction

Treatment with anti-resorption and anti-angiogenic drugs associated with an exposure of jaw bone or fistula for more than eight weeks, and in the absence of radiation exposure of head and neck, defines the diagnosis of medication-related osteonecrosis of the jaws (MRONJ) [1]. Until today, bisphosphonates [2], denosumab [3], and antiangiogenic drugs such as sunitinib, bevacizumab, or aflibercept [4] have been related with MRONJ. Bisphosphonates are taken into the osteoclasts and manifest a long-term antiresorptive action [5]. Denosumab inhibits the receptor activator of nuclear factor-κB binding (RANKL) and the receptor activator of nuclear factor-κB (RANK). This RANKL-RANK complex is essential in osteoclast-mediated bone resorption, and its inhibition decreases the bone turnover, promoting the risk to induce MRONJ [6]. Its half-life is 25 to 32 days [7]. Antiangiogenic drugs inhibit the Vascular Endothelial Growth Factor (VEGF) (and this is the case of Bevacizumab), inhibit tyrosine-kinase (i.e., by Sunitinib), or the mammalian target of Rapamycin (in the case of Sirolimus or Everolimus) [8].

The first case related to bisphosphonates treatment was described in 2003 [9], while the first one implying denosumab, with a similar action mechanism, was reported in 2010 [10]. The first case report associating bevacizumab with the incidence of osteonecrosis of the jaws was made in 2008 [11]. Prevalence of MRONJ after bisphosphonates has been reported in different studies, with a wide range of 0% to 27.5% [12]. In contrast, the risk of developing MRONJ induced by denosumab ranges only up to 2% [13]. This slow incidence of MRONJ after a denosumab treatment could be explained by its short life-time and lack of bone binding. Its main action is related to the decrease of the differentiation of osteoclasts [14,15]. Anti-angiogenic inhibitors that have been widely used in the treatment of ovarian cancer, metastatic renal cell cancer, breast cancer, colorectal cancer, non-small-cell lung cancer, and glioblastoma multiforme [16] induce bone exposure associated with pain in a large range, of up to 91.43% [17].

The 2014 position paper of The American Association of Oral and Maxillofacial Surgeons (AAOMS) has classified MRONJ into five stages [18]. The first “at risk” stage includes patients with no apparent necrotic bone who have been treated with oral and intravenous bisphosphonates. Even without a clinical evidence of the disease, patients in stage 0 draw attention because of the fact that up to 50% of them progress in the next stages of this condition. The clinical aspect of exposed or necrotic bone, or of fistula frames brings the case to a higher level in the classification, in stages 1, 2, or 3. The clinical signs of the MRONJ can occur both spontaneously (caused by a dental or periodontal infection) or induced by a local trauma [19,20].

The management strategies for patients who are candidates for antiresorptive or anti-angiogenic medication start with a dental screening followed by a convenient treatment [1]. The evaluation of a patient who may undergo such a treatment should include a panoramic radiograph with the identification of any active or potentially active oral infections. The treatment plan aims to eliminate all acute and potential infection areas [21]. This preventive dental program could reduce the risk of osteonecrosis of the jaws with up to 50% [22]. Periodontal diseases and chronic dental illnesses, for example, induce bacterial infections and therefore a reaction of the immune system [23]. This inflammatory response occurs throughout a significant increase in the levels of IL-6 and urokinase-type plasminogen activator receptor (suPAR) [24,25]. Based on the availability of reliable salivary biomarkers for patients undergoing anticoagulants and antiresorptive medication, an early diagnosis of MRONJ could make a major contribution to the correct medical management of these patients, reducing their morbidities and clinical conditions [25,26].

The 2014 consensus conference of AAOMS suggested that a conservative approach should be preferred for initial stages (0 and 1), while a surgical debridement should be added to advanced ones [1,18]. Thus, the most debated topic on MRONJ is its therapy, as there are no definitive guidelines yet [27].

Therefore, the aim of the present study was to explore and develop different strategies (i.e., for different stages of MRONJ), starting from the experience of different existing approaches, and to further demonstrate their effectiveness.

Today, a stage-dependent approach does not currently represent the main guide in the treatment of MRONJ-affected patients [28], and this is one of the main issues the present study addresses. A recent review concluded that surgical therapy compared with nonsurgical therapy has been associated with disease resolution [29]. The reported success rate has ranged from 34.5% to 90% [30,31]. 

To improve the treatment results, combined therapies using high-end technologies devices have been applied [30], and this is the strategic approach that we considered, as well. As an additional component during the surgery, plasma rich fibrin (PRF) has increased the fibroblasts and osteoblasts migration, proliferation and viability, with positive effect in bisphosphonate-related osteonecrosis of the jaws (BRONJ) treatment [31]. The exposed bone offers a strong microbial support that might increase and maintain the inflammatory and necrosis of tissues [32]. Besides bone debridement, a positive outcome also implies bone decontamination. Because of its cavitation effect, with direct killing result on bacteria, piezo-surgery devices could be applicable for necrotic bone removal [33].

Photo-biomodulation (PBM) has been applied in many fields of medicine for its analgesic and anti-inflammatory effect [34]. It is also a tissue healing and repair accelerator [35,36,37,38]. The increased secretion of growth factors and the faster differentiation of stem cells promoted by PBM during bone regeneration have been already proven [39,40,41]. During PBM, cytochrome C oxidase (unit four in the mitochondrial respiratory chain) is stimulated. This enzyme activation, sensitive to light, induces an increase in cell proliferation, migration, differentiation, and metabolic activity [38]. The positive effect of PBM in association with conservative or surgical treatment of MRONJ has been already highlighted [42,43,44,45]. As PBM improves wound healing and modulates cell metabolism, it is already assessed as a complementary therapy in BRONJ [44]. Pre-conditioning tissues using PBM are used in pathological conditions where tissue damage may be expected. Such an effect of PBM on local tissues improves healing after surgical procedures [46]. 

Considering the above, the aim of the present study is to apply and then to analyze the results of a composed and stage-approach therapy combining antibiotherapy, surgical treatment, and PBM for the prevention and treatment of MRONJ lesions. The rationale of this work is given by the expected increase in MRONJ cases in the future, because of the cumulative effect of bisphosphonates in bone and the broad targeting of antiangiogenic drugs in many severe human diseases. The null hypothesis of the present study is that the development of combined, MRONJ stage-related therapy protocols for this condition is the most appropriate approach.

## 2. Materials and Methods

A prospective monocentric observational study has been carried out between March 2018 and January 2020 at the Department of Oral and Maxillofacial Surgery, School of Dental Medicine of the “Victor Babes” University of Medicine and Farmacy of Timisoara. This study was approved by the Ethics Commission for Scientific Research, following the Ethical protocol of the University, with the CECS Approval nr. 09/02 March 2018 (approved date: 2 March 2018), and it was carried out according to the Declaration of Helsinki. An informed consent was submitted to all the enrolled patients. A total of 241 patients previously exposed to one of the antiresorptive or anti-angiogenic drugs, therefore diagnosed in different clinical stages of the condition, were referred to the above department for oral surgery or treatment of oral manifestations of MRONJ. Clinical diagnosis was supplemented with orthopantomography for the first evaluation, and with cone beam computed tomography (CBCT) to establish the extension of bony lesions. Each patient’s affiliation at a certain stage of disease was made following the clinical criteria of AAMOS-2014 [1,18]. Surgical procedures were performed by certified oral and maxillofacial surgeons from the clinic department. For all the 241 patients enrolled in the study, a professional dental cleaning-full mouth disinfection was performed before starting the procedures. Chlorhexidine solution 0.2% was indicated for the 6-week perioperative period.

The proposed and applied therapy procedures were different for each stage of MRONJ, as follows:

(a) Patients staged “at-risk” for MRONJ and referred for tooth extraction required oral surgery procedures, but they had no symptoms and oral signs of MRONJ. They received Amoxicillin/Clavulanic acid 1 g/12 h or, in case of drug allergy, 600 mg Clindamycin orally, 3 days before and 7 days after extraction, 2 times a day. Dental extractions were carried out with minimum trauma. After curettage and lavage with saline solution, a crossed horizontal external suture was applied to limit the socket entrance and to cover the bony margins. In the next step, near infrared (NIR) InGaAsP Diode laser (EPIC X™, BIOLASE^®^, Foothill Ranch, CA, USA), with a center wavelength of 940 nm, was used to photo-biomodulate the socket from the buccal, as well as from the lingual side, perpendicular to the surface. For delivering the laser irradiation, the PBM tissue handpiece by BIOLASE^®^ was used, with a laser beam diameter of 9 mm and with an irradiation area of 0.635 cm^2^. PBM was performed using the following parameters and settings: power 100 mW, power density 157.4 W/cm^2^, in continuous mode, irradiation time 40 s on each side, energy 8 J per each session, energy density 3.937 J/cm^2^ (in non-contact mode, 1 mm from the tissue surface). After tooth extraction, the PBM was performed at 24 h, 48 h, 72 h, day 4, day 5, day 6, and day 7, as well as 3 times/week for the following 2 weeks after surgery. The sutures were removed 10 days after the surgery. Mouth rinsing with 0.12% chlorhexidine digluconate was prescribed for 6 weeks.
(b) The patients in stage 0 of MRONJ received only antibiotic treatment and PBM, with no surgery. The treatment was received for 14 days: Amoxicillin/Clavulanic acid 1 g every 12 h or, in case of drug allergy, 600 mg Clindamycin orally, twice a day. To reduce the local inflammation associated with pain, PBM was performed during the 7 consecutive days, followed by other 6 sessions of laser irradiation distributed in the following 2 weeks.
(c) For all the patients in stages 1, 2, and 3 of MRONJ, the treatment protocol was perioperative antibiotic, preoperative PBM, and surgery. The antibiotic treatment was prescribed 3 days before surgery, as well as 14 days after the surgery. To increase the healing by pre-conditioning the tissues, PBM was applied 3 consecutive days before surgery, with the same parameters mentioned above. The necrotic bone was removed using an ultrasonic device “Piezotome II” (Satelec-ACTEON, France). After the local infiltration of anesthetic solution without vasoconstrictors, a flap was raised to have direct access to the necrotic bone. The procedures required to eliminate the damaged bone (i.e., debridement, sequestrectomy, block resection, and osteoplasty) were performed in accordance with the preoperative radiological findings and with the intraoperative bleeding occurrence within the remaining bone.

From each patient, prior to anesthesia administration, blood samples were collected in 10 mL collection tubes without anticoagulant content. The blood was immediately centrifuged (Intra-Spin^®^ EBA 200, Intra-Lock System, Beverly, MA, USA), with a force of approximately 400 g for 12 min, at 2700 rpm. PRF membranes, obtained after shaping with PRF-Box^®^ (Intra-Lock System, Boca Raton, FL, USA), completely covered the bone. A tension-free primary closure was achieved in all cases. A soft diet was prescribed for two weeks, and topic daily mouth rinsing with 0.12% chlorhexidine digluconate was prescribed. Patients were scheduled for periodical clinical follow-up, for at least 6 months after the treatment.

Examples of the evolution of each of the above cases are presented in the following section.

Patients in an “at risk” stage of MRONJ, who required oral surgery procedures, were evaluated 8 weeks after the surgical time and reported as healed when the soft tissue has totally covered the sockets. A complete mucosal healing without any clinical symptoms recorded 8 weeks after the treatment was considered as a disease resolution. For patients in advanced stages of MRONJ, a downscaling to stage 1 was also considered a treatment success. However, as discussed further on in the paper, this inclusion can be subject for future debate in the community.

## 3. Results

Two hundred forty-one patients exposed to one of the drugs associated with MRONJ and diagnosed with one of its different clinical stages were referred to the Department of Oral and Maxillofacial Surgery for routine dental extractions. They required oral surgeries or shown signs and symptoms of the disease. The following information was gathered from each patient: age, gender, pathology that required antiresorptive and anti-angiogenic medication therapy, type of administrated medication, route of administration, time of use, MRONJ stages following AAMOS criteria, location of osteonecrosis, type of applied therapy, and clinical results after treatment. The patients’ age varied between 46 and 79 years; the mean age was 67.7 years. From the 241 patients, 184 were female (76.34%) and 57 were male (23.65%). The most common co-morbidity with indication of antiresorptive was osteoporosis.

From all the patients, 143 of them (i.e., 59.33%), only female, were treated for osteoporosis; for them, the route of administration was oral. The indicated medication was Ibandronat, 150 mg per month; Alendronat, 280 mg per month; Risendronat, 75 mg per month. This treatment lasted between three to five years. 

All the other patients, *n* = 98 (*n* = 41 female and *n* = 57 male) were treated for an underlying malignant disease. From these patients, *n* = 94 were treated with zoledronic acid, 4 mg/intravenous, monthly, for a period of 12 to 84 months; *n* = 4 patients were treated with anti-angiogenic medication, Sunitinib, 50 mg per day, for a period of 12 to 36 months, combined with zoledronic acid for the same time.

According to the AAOMS-defined stages [18], *n* = 84 patients belonged to the “at risk” stage, *n* = 49 patients belonged to stage 0 of MRONJ, *n* = 10 to stage 1, *n* = 91 to stage 2, and *n* = 7 patients to stage 3.

**Figure 1:**
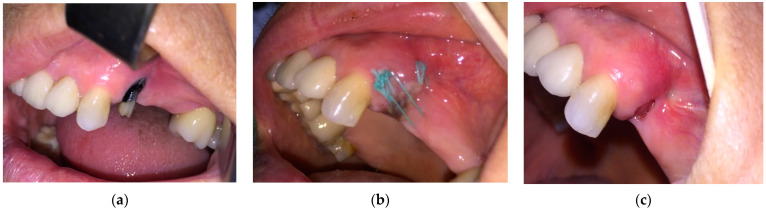
(**a**) Patient with the 1st upper premolar indicated for extraction; (**b**) 10 days after extraction; (**c**) complete healing after 8 weeks.

**Figure 2:**
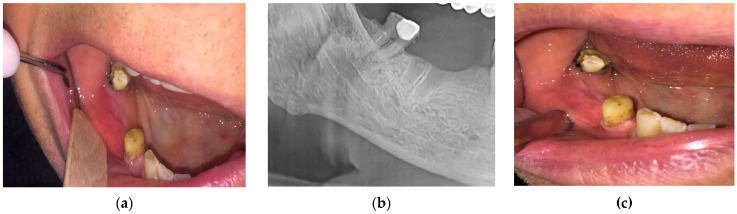
(**a**) Patient diagnosed with congestion and edema of oral mucosa (4th quadrant) in stage 0 of medication-related osteonecrosis of the jaws (MRONJ); (**b**) panoramic radiography showing no alveolus remodeling 5 months after the extraction performed in the 4th quadrant; (**c**) complete remission of pain, edema, and mucosal inflammation after antibiotics therapy and photo-biomodulation (PBM).

**Figure 3:**
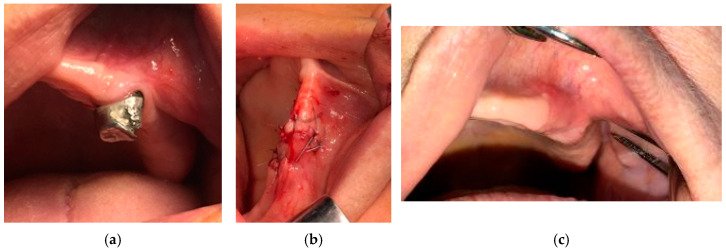
(**a**) Patient in stage 0 of MRONJ, initial presentation; (**b**) during the surgical healing, after the tooth extraction; (**c**) 3 months after the surgery.

The results obtained for the different stages were as follows:

(a) For the *n* = 84 patients from the “at risk” group, the extractions were performed under the protocol mentioned above. The healing was complete with a spontaneous bone coverage in all cases. An example of the evolution of such a case is presented in Figure 1.
(b) For the *n* = 49 patients belonging to stage 0 of MRONJ, a pain reduction and a decrease of the mucosal inflammation was obtained in all cases. Two examples of the evolution of such cases are presented in Figure 2 and Figure 3.
(c) The surgical therapy outcome was analyzed for *n* = 108 patients with MRONJ in stages 1, 2, and 3, treated with intravenous BP and with oral anti-angiogenic medication. The most common initiating factor was teeth extraction. Most lesions (for *n* = 82, i.e., for 75.92% of the patients) were placed in the mandible, while a certain number (for *n* = 26, i.e., for 24.08% of the patients) were placed in the maxilla. The same protocol was applied to all the patients proposed for surgery. A complete disease resolution was obtained in 99 cases (all in stages 1 or 2 of the disease) from the total of 108 cases for which the healing was obtained with the first surgical treatment. In *n* = 9 cases, *n* = 2 in stage 2 and *n* = 7 in stage 3 of MRONJ, a downscaling to stage 1 was obtained, with a significant increase in the quality of life. The specific feature of these two patients in stage 2 who were downscaled is that they were treated for underlying malignant disease with zoledronic acid intravenous associated with an oral treatment with Sunitinib. In a case belonging to stage 3, the recurrence of infection occurred four months after the initial treatment. Thus, for the patients in stage 3 (*n* = 7), a downscaling to stage 1 was obtained for six cases, corresponding to a healing rate of 85.71%.

Therefore, the percentage of patients with MRONJ in stages 1, 2, and 3 who had a complete healing is 91.66% (while for 7.4% a downscaling to stage 1 was obtained). This brings the overall healing rate (of all 241 patients) to 99.59% if downscaling to stage 1 is also considered a success of the therapy.

As a remark, because of the complete healing rate of patients in “at risk”, 0, and 1 stage of MRONJ, one may say that the proportions of such patients considered in the present study does not influence the statistic relevance on the study.

**Figure 4:**
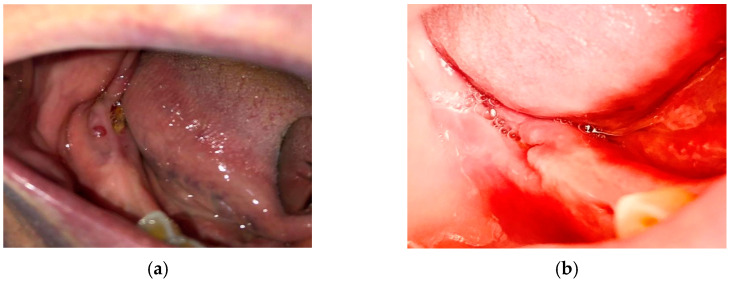
(**a**) Posterior mandible of a patient with a stage 1 of MRONJ; (**b**) complete healing, 3 months after surgery, antibiotic therapy, and PBM.

**Figure 5:**
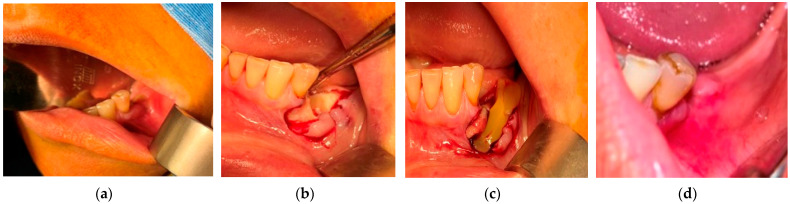
(**a**) Patient in stage 2 of MRONJ at initial presentation; she was downscaled at stage 1 after PBM and antibiotherapy and was further on proposed for surgery; (**b**) performed surgery, with necrotic bone removal; (**c**) plasma rich fibrin (PRF) was applied in 2 layers; (**d**) complete bone coverage after the treatment.

**Figure 6:**
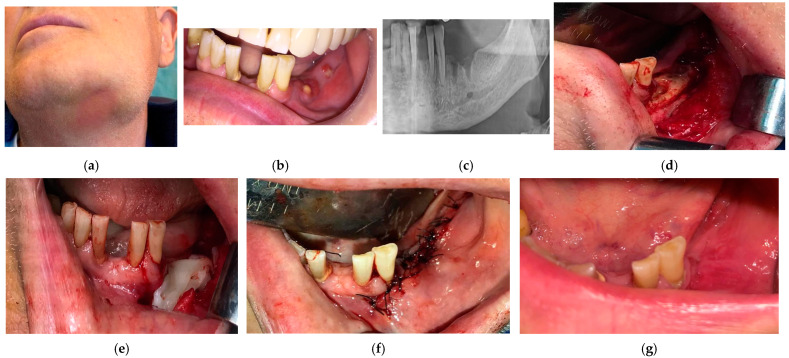
(**a**) Patient in stage 3 of MRONJ with infection extended to the inferior border of mandible-extraoral view; (**b**) intraoral view at the initial presentation of the patient; (**c**) mandible bone showing a sclerotic structure; (**d**) performed surgery, with necrotic block resection; (**e**) PRF membranes covering completely the bone in 2 layers; (**f**) tension-free closure of the surgical wound; (**g**) complete healing achieved 6 months after the end of the treatment.

Examples of the evolution of patients with MRONJ in stages 1, 2, and 3 are presented in Figure 4, Figure 5 and Figure 6, respectively.

## 4. Discussion

We reported our experience with a multiplex and stage-approach therapy integrating antibiotherapy and PBM in a precise sequence for prevention of MRONJ; also, with an additional minimal invasive piezosurgery treatment associated with PRF in cases of established conditions. Treatment of MRONJ is challenging for both oral and maxillofacial fields. No consensus has been established so far regarding the best treatment option throughout all the stages of the disease, but the main objectives have been closely followed by every proposed protocol: infection control, tissue healing support, and slowing the course of the disease [1,18].

There are several methods of treatment for MRONJ already described in the literature. The treatment directions could be summarized in medication treatment [47,48], conventional [49], laser [50] or ultrasonic piezoelectric surgery [51,52], biostimulator treatment [53,54], or combined therapies [4]. The effectiveness of these treatment methods shows ample variation in results. Oral antibiotic therapy may have a finite effect on the bacterial population associated with MRONJ [55], therefore a conservative antibiotic treatment applied as a single tool method has been successful for only 23% of the patients [56]. The surgically-treated cases associated with a preoperative antibiotic therapy was demonstrated to have a complete healing in 47% to 87% of the cases [57], although a recent retrospective study showed that this limit can be pushed over 90% [58]. The microbial aggregation that develops between the hydroxyapatite and bisphosphonates could lead to progressive inflammation and necrosis of bone and surrounding soft tissue [32,59,60]. 

Referring to these aspects, the cavitation effect that takes place during the ultrasonic vibration proved able to decrease the microbial mass around the infected bone, acting alongside the antibiotic effect [61]. Furthermore, the controlled and selective cuts performed by the piezosurgery tool induce a rapid accumulation of bone morphogenetic protein, with anti-inflammatory and bone remodeling effect. Data gathered from the literature show the effectiveness of surgery using piezosurgery devices associated with antibiotic in patients with MRONJ [34,60]. 

Autologous platelet concentrates have been used in the oral and maxillofacial field for more than 20 years, and in 2007 they have been used for the first time in MRONJ, because of their potential of cell proliferation and osteogenic differentiation [62]. There are several reports with positive outcomes related to the use of different autologous platelet concentrates, especially PRF in MRONJ [63]. PRF has the advantage of being an easy, ready to use and inexpensive preparation method. Applied in cases that undergo a zoledronic acid treatment, PRF significantly enhances the proliferation of fibroblasts and osteoblasts [31]. The direct clinical effect is an early epithelization of the surgical sites, due to its property to be released at least one week, and at most 28 days during the healing process [64,65,66,67].

The biostimulatory treatment, especially PBM, has been introduced as a therapy method about 50 years ago to increase the healing potential of tissues, as well as to relieve pain, inflammation, and swelling. More than this, PBM can be applied when tissue damage may be expected, specifically before surgery, with the scope to induce a protective response against the later scheduled medical act [4]. Following this approach, we applied PBM three days before surgery to reduce inflammation and pain, and to pre-condition the oral mucosa.

Referring to the protocol applied in this study (and described in Section 2), it has been widely demonstrated that diode lasers with a wavelength in the 655–980 nm interval accelerate wound healing by stimulating natural biological processes such as angiogenesis and release of growth factors [68]. Our irradiation protocol has been linked to the natural healing process after oral surgery, in accordance with the current literature. Thus, a surgical wound is followed by a degree of inflammation with its highest level occurring after 24 and 48 h [69]. This is the reason why we applied PBM daily in the first week after surgery. Moreover, the wound healing after extraction requires various periods of time that can last up to 24 weeks for healthy patients [70]. Histological improvements of soft tissue and bone regeneration of socket after extraction have been demonstrated in a human study applying daily PBM protocol for the first seven days after extraction [71]. Therefore, patients affected by MRONJ received weekly applications of PBM for the first six weeks, with the possibility of additional applications until a complete mucosal healing [72].

In the proposed procedures, we applied PBM in all “at risk” cases by following a predefined irradiation protocol. In this way, the patients were supervised for three weeks after the extractions by applying the PBM during the period of soft and bone tissue remodeling of the socket. The healing was complete for all *n* = 84 patients in this stage, with a 100% spontaneous bone coverage. At this stage, the PBM therapy protocol proved to be a useful adjuvant light application procedure that prevents MRONJ from developing. A limitation of this part of the present observational study regarding patients in “at risk” stage is that all these patients have been under treatment with oral bisphosphonates, which lowered the risk for MRONJ [15]. The use of PBM proved extremely useful in all *n* = 49 cases in stage 0, where inflammation and pain reduction were obtained, downscaling these patients to an asymptomatic condition. In the same time, the risk of disease progression was reduced.

From the *n* = 108 patients proposed for surgery, a complete disease resolution was obtained in 99 cases (i.e., 91.66%), all of them in stages 1 and 2 of the disease. In *n* = 9 cases (*n* = 2 in stage 2 and *n* = 7 in stage 3 of MRONJ), a downscaling to stage 1 was obtained. 

In our selected cases, the surgical approach, preceded and followed by laser PBM, to pre-condition the tissues and to support the healing, respectively, as well as associated with antibiotic therapy, led to a high overall success rate of 96.68% when the “success” rate includes only a complete healing of the patients, and of 99.59% when a downscaling to stage 1 (i.e., of patients with stages 2 and 3 of MRONJ) is considered as a success of the therapy, as well. 

Therefore, the developed protocol provided a higher healing rate compared to the surgical treatment alone or associated with antibiotic perioperatively, where a rate between 47% and 87% has been previously reported [57], while a retrospective study regarding 116 AAOMS patients with MRONJ stages 1, 2, or 3 has indicated a 93.97% success rate [58]. 

A further debate in the community may target the inclusion in the success rate of the downscaled cases, as they still correspond to patients that have stage 1 of MRONJ, therefore have a condition, although their quality of life has improved. This is the reason why we chose to provide the results of the treatments in two different ways, including the downscaling to stage 1 as a treatment success (when we may report a 99.59% healing rate) and, in contrast, including only the patients completely healed in the success rate, which would bring it only to 96.68%, which is somehow more realistic. Considering this aspect may be a challenge for the community, as well, besides developing (more) efficient, combined, and stage-approach therapies.

Such an approach is confirmed by recent studies, which point out to the better effectiveness of combining different techniques for treating MRONJ [73], but also to the necessity to diagnose it early and to treat it efficiently [74]. Other recent aspects of interest refer to telemedicine approaches of such conditions in pandemic situations [75].

Besides the techniques utilized in this study, several others are of interest, and they are subject of future work for our groups. Fluorescence-guided surgery is such a reliable and predictable method for the diagnosis of bone margins. Using the erbium-doped yttrium aluminum garnet (Er: YAG) laser is advantageous in bone ablation because of a high absorption rate of light by the hard tissue components. It also stimulates the secretion of platelet-derived growth factor in osteotomy sites and has bactericidal effect against Actinomyces and anaerobes [76]. Applying such a technique can be subject of future work.

Furthermore, a combination of antimicrobial photodynamic therapy (aPDT) and PBM has the goal to reduce the use of antibiotics in patients suffered from MRONJ [77]. While this has not been part of our protocol so far, it may also be subject of future investigations.

For the surgical treatment to be efficient, it aims at a necrotic bone removal with a complete mucosal healing. To achieve this, a correct delineation of necrotic bone during resection is the major demand [18]. Thus, the most challenging step during the surgical treatment is to define the limit of resection. Bone texture and color, as well as bleeding margins are subjectively appreciated by the surgeon during a conventional surgical treatment [78]. The surgical experience linked to imaging investigations are used to remove the bone with signs of necrosis. As a support of the practical field, autofluorescence-guided surgery (using the Velscope device) represents a reliable approach which can improve the treatment outcomes by delineating the necrotic bone [79,80,81]. Although the use of the Velscope in guiding the necrotic bone resection shows to be helpful, the postoperative results assessed by mucosal healing and quality of life indicate to be similar to conventional surgery [82].

Considering the above, we must highlight that our proposed prevention or treatment protocols are not a definitive solution but an exploration of a combination of some of the more at-hand therapies available today. Therefore, it is by all means prone to further optimization, considering both other existing or further developed techniques.

Furthermore, besides different therapeutic approaches, one must highlight the importance of early diagnosis of MRONJ. In this respect, the availability of reliable salivary biomarkers, for example, could improve the health maintenance in order to reduce the alveolar bone loss and the inflammatory process [25,26]. 

## 5. Conclusions

The reported results indicated that MRONJ can be prevented by an early dental examination and an adequate preventive treatment. The clinical outcome of the present study indicated that patients with MRONJ in almost all stages of the disease can benefit from an association of different methods (i.e., antibiotherapy, surgical treatment, and PBM). Thus, the obtained clinical results have been superior compared with classical therapies. Therefore, the null hypothesis of the study was confirmed: the development of combined, MRONJ stage-related therapy protocols for this condition (such as those we have worked on) is the most appropriate approach. However, as highlighted in the Discussion section, numerous methods we have not (yet) explored can be considered, and protocols can definitely be improved for each stage of MRONJ, including for its early diagnosis and prevention.

Such treatment protocols can contribute to approaching MRONJ cases, considering the obtained stabilization of the surgical sites and the demonstrated low recurrences rate in the present work. The clinical impact of such studies and methods development is given by the increasing number of MRONJ cases expected in the future. Such problems are caused by the cumulative effect of bisphosphonates in bone and the broad targeting of antiangiogenic drugs in many severe human diseases. Therefore, a well-established management of these patients, with rigorous protocols of prevention and treatment, must be developed and applied. Moreover, a conceptual clarification regarding the inclusion of the downscaled patients (from stage 2 or 3 of MRONJ to stage 1) in the success rate of the treatments may also be beneficial.

## Data Availability

The data on the patients included in this clinical study are not publicly available due to ethical and privacy reasons.
